# A New Method of Administering Koosso

**Published:** 1880-01

**Authors:** 


### A NEW METHOD OF ADMINISTERING KOOSSO.

Of all the remedies for tape worm, none is more certain or efficient than Koosso, and many efforts have been made to bring it into such pharmaceutical shape that, while its properties as a tonicide remain unimpaired, it might be administered without repugnance. Dr. Corre, some years ago, proposed the following method which has been successfully used in many cases: Onehalf ounce of fresh-powdered koosso is treated with one ounce of hot castor oil, and afterward with two ounces of boiling water by displacement; express, and by means of the yolk of an egg combine the two, percolate into an emulsion, and add forty drops of sulphuric ether, flavoring with some aromatic oil.

This is to be taken at one dose early in the morning, after a previous fast of about eighteen hours. The worm is usually expelled dead after six or eight hours.

